# Unilateral Biportal Endoscopic Decompression for Degenerative Lumbar Spinal Stenosis Under Local Anesthesia in Elderly Patients with Medical Comorbidities

**DOI:** 10.1111/os.70114

**Published:** 2025-07-15

**Authors:** Haining Tan, Yuquan Liu, Guangpeng Li, Lingjia Yu, Haibo Sun, Bin Zhu, Qi Fei, Yong Yang, Yuan‐Shun Lo, Xiang Li

**Affiliations:** ^1^ Department of Orthopedics, Beijing Friendship Hospital Capital Medical University Beijing China; ^2^ Division of Spine Surgery, Department of Orthopedic Surgery, China Medical University Beigang Hospital China Medical University Yunlin Taiwan; ^3^ Division of Spine Surgery, Department of Orthopedic Surgery, China Medical University Hospital China Medical University Taichung Taiwan; ^4^ Spine Center China Medical University Hospital Taichung City Taiwan

**Keywords:** elderly spine surgery, local anesthesia, lumbar spinal stenosis, medical comorbidity, unilateral biportal endoscopy

## Abstract

**Objective:**

Conventional Unilateral Biportal Endoscopic (UBE) surgery usually requires general anesthesia (GA), which introduces additional risks to patients with significant medical comorbidities. This article explores the use of UBE decompression under local anesthesia (LA) in elderly patients with severe medical comorbidities treated at our institution, providing valuable clinical insights for the application of this technique.

**Methods:**

A retrospective analysis was conducted on patients clinically diagnosed with lumbar spinal stenosis (LSS) at our center between November 2021 and March 2024, who underwent UBE decompression surgery under local LA. The data collected included demographics, visual analog scale (VAS) scores for leg pain, oswestry disability index (ODI), and modified Macnab grades. The UBE decompression procedure was divided into seven key steps, and intraoperative pain and the effectiveness of LA were assessed using patient self‐reported VAS scores at each step. Data comparisons between the preoperative, postoperative, and follow‐up time points were conducted using paired sample *t*‐tests.

**Results:**

Eighteen patients (5 males and 13 females) with an average age of 77.1 ± 5.0 years were included in the study, with 83.3% (15 patients) having medical comorbidities. The average follow‐up period was 14.8 ± 7.9 months. At 3 months postoperative and final follow‐up, both VAS scores for leg pain (*p* < 0.001) and ODI scores (*p* < 0.001) showed significant improvement. According to the modified Macnab criteria, outcomes were rated as excellent in 13 patients (72.2%), good in one (5.6%), fair in two (11.1%), and poor in one (5.6%), yielding an excellent‐good rate of 77.8%. None of the patients voluntarily requested surgery termination because of unbearable intraoperative pain.

**Conclusions:**

For elderly patients with medical comorbidities, UBE decompression under LA is a viable and effective treatment option, yielding favorable clinical outcomes.

## Graphical abstract

For elderly patients with LSS who are intolerant to general anesthesia, UBE decompression under local anesthesia is feasible. Satisfactory clinical outcomes can be achieved by employing the *SafetyNet*‐guided decompression strategy during UBE surgery under local anesthesia. UBE decompression under local anesthesia is a potential alternative treatment for elderly patients with LSS and significant medical comorbidities.

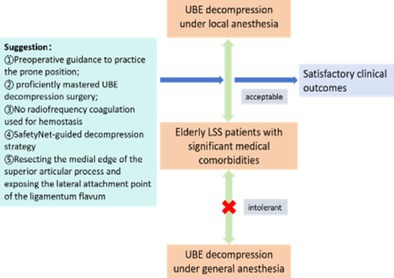

## 1 Introduction

Lumbar spinal stenosis (LSS) is characterized by narrowing of the spinal canal in the lumbar spine, leading to compression of the spinal cord and nerve roots. It can result in lower back pain, radiating leg pain, and neurogenic claudication, and has been reported to be highly prevalent in elder patients [1, 2]. As some studies have reported, patients with LSS aged over 70 or 80 years could benefit from decompressive operation [3, 4]. However, some studies have also shown that advanced age and multiple comorbidities are risk factors for perioperative complications in lumbar spine surgery [5, 6, 7].

In recent years, unilateral biportal endoscopic (UBE) decompression has been increasingly used to treat patients with LSS who present with severe symptoms and do not respond to conservative treatments. Compared with traditional open laminectomy and other minimally invasive techniques, UBE has shown promising functional and radiological outcomes, with advantages such as lower complication rate, reduced blood loss, shorter hospital stays, and faster recovery [8, 9, 10, 11, 12, 13, 14].

General anesthesia (GA) is typically required in conventional UBE surgery. However, it may increase the risk of cardiac, cerebral, and respiratory complications during the perioperative period, particularly in elderly patients with multiple comorbidities [15, 16]. As an alternative anesthesia, spinal anesthesia (SA) is feasible for elderly patients with multiple comorbidities who are unsuitable for GA. There have been some reports in the literature on successful lumbar spine surgeries performed under SA that achieved satisfactory clinical outcomes [17, 18, 19, 20, 21]. However, for UBE technique, SA still carries potential risks, especially for increased intracranial pressure, subsequent seizures and even death [22]. While, local anesthesia (LA) has been successfully applied in the uniportal endoscopic decompression technique for treating LSS [23, 24], suggesting its potential feasibility in UBE procedures.

To the best of our knowledge, only a limited number of studies have reported UBE under total LA for degenerative lumbar diseases. This study aimed to outline the technical details of UBE decompression surgery under LA and evaluate its feasibility and preliminary outcomes for treating LSS.

## 2 Methods

### 2.1 Subjects

We retrospectively reviewed the medical records of patients with LSS who underwent UBE decompression under LA at our spine center between November 2021 and March 2024. The inclusion criteria were as follows: [1] diagnosis of LSS at single level, [2] primary symptoms of radiating leg pain and/or neurogenic intermittent claudication, and at least 3 months of ineffective conservative treatment. The exclusion criteria were as follows: [1] age < 18 years; [2] presence of a tumor, infection, or trauma in the lumbar spine; [3] presence of scoliosis, ankylosing spondylitis, or other spinal deformities; [4] lumbar spondylolisthesis greater than Grade II; [5] history of surgery at the same level; [6] incomplete medical records; and [7] follow up less than 6 months.

### 2.2 Surgical Procedure

1. *Positioning and surgical site preparation*: All procedures were conducted with the patient in the prone position with the lumbar spine slightly flexed. The surgical level was confirmed under fluoroscopic guidance, after which the lumbosacral area was prepared and sterilized.
2. *Incision planning*: Two transverse incisions, each 1 cm in length, were planned approximately 1.5 cm above and below the target site. The incisions were positioned at the intersection of the lower margin of the upper vertebral lamina and midline of the inferior articular process of the lower vertebra.
3. *Local anesthesia*: All procedures were performed under intravenous administration of 5 μg sufentanil and local anesthesia using a mixture of 1% ropivacaine (10 mL), 2% lidocaine (15 mL), and 0.9% normal saline (20 mL). Layer‐by‐layer infiltration anesthesia was applied to the surgical area, including the skin, subcutaneous tissue, fascia, muscles, lamina, periosteum of the spinous process, and facet joint capsule.
4. *Establishment of working portals*: The locations of the working portals were confirmed under fluoroscopic guidance. The muscles were then separated using a blunt dissector and an electrocautery device. The cranial portal was designated for the 0° endoscope with a continuous irrigation system, while the caudal portal was used for decompression.
5. *Decompression*: Bony decompression was performed using a 4 mm arthroscopic burr, Kerrison rongeur, traditional osteotome, and an ultrasonic bone scalpel. After confirming complete detachment from both the dura and lamina, the ligamentum flavum was removed using Kerrison rongeur or pituitary forceps. Decompression was considered complete once normal respiration‐induced pulsation of the dura and the nerve roots was observed. A “*SafetyNet*”‐guided seven‐step decompression strategy was employed in our case series:

*Step 1*: The lower edge of the upper lamina was ground to expose the cephalic end of the ligamentum flavum.

*Step 2*: Exposing *SafetyNet*, an anatomical landmark located at the lower edge of the inferior articular process and at the base of the superior articular process.

*Step 3*: The medial edge of the inferior facet joint was removed from *SafetyNet*.

*Step 4*: Separating the insertion point of the ligamentum flavum in the *SafetyNet* area.

*Step 5*: Separating the distal insertion point of the ligamentum flavum from *SafetyNet*.

*Step 6*: The medial edge of the superior articular process and the lateral insertion point of the ligamentum flavum were removed from *SafetyNet*.

*Step 7*: Removing the ipsilateral ligamentum flavum from the midline cleft.

**FIGURE 1:**
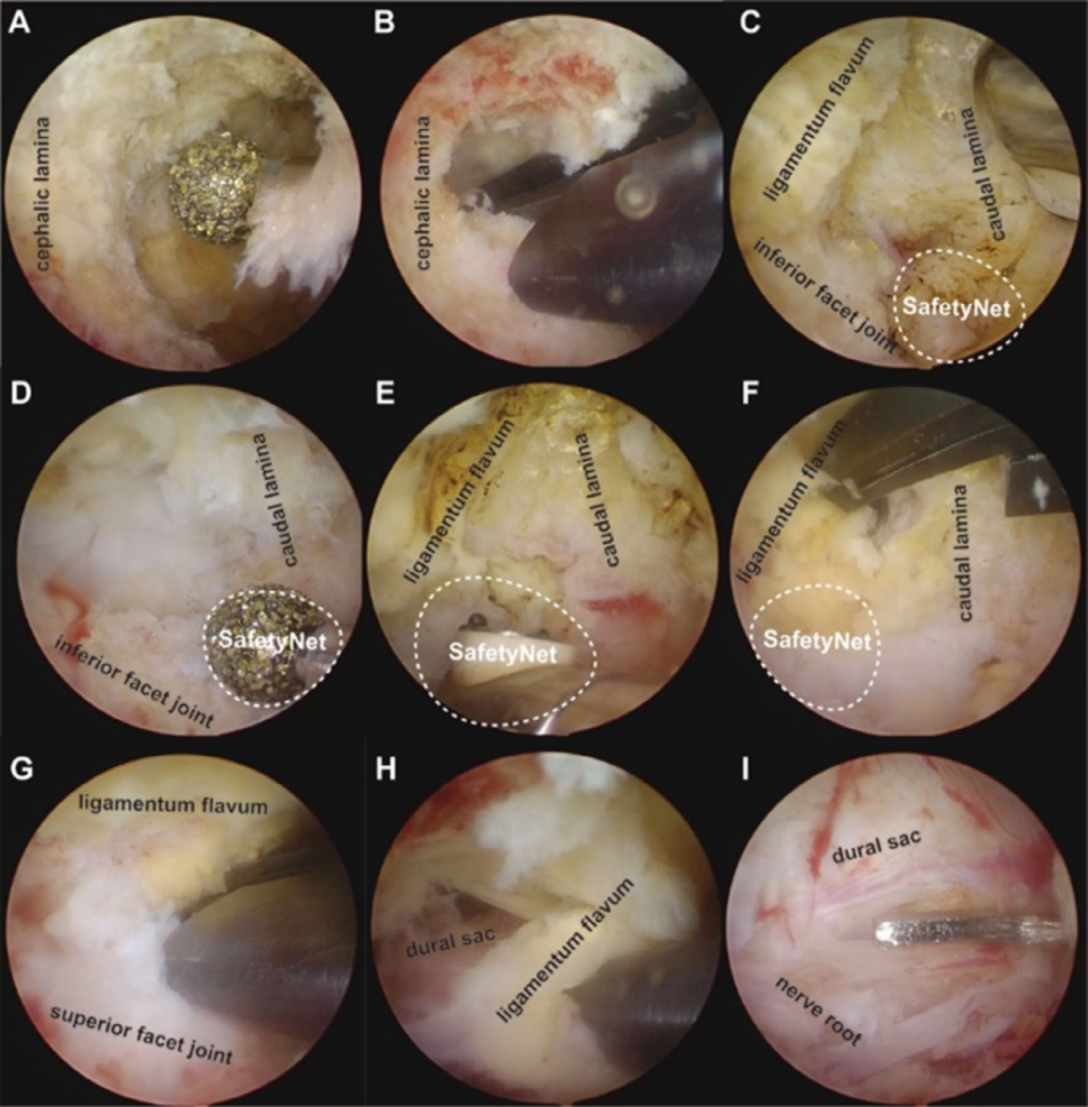
“*SafetyNet*”‐guided seven‐step decompression: (A, B) resecting the lower edge of the upper lamina and exposing the cephalic end of LF, (C) exposing *SafetyNet*, (D) removing the medial edge of the inferior facet joint, (E) separating LF in the *SafetyNet* area, (F) separating the distal insertion point of LF, (G) removing the medial edge of the superior articular process and the lateral insertion point of LF, (H, I) removing LF from the midline cleft and completing decompression. LF, ligamentum flavum.

**FIGURE 2:**
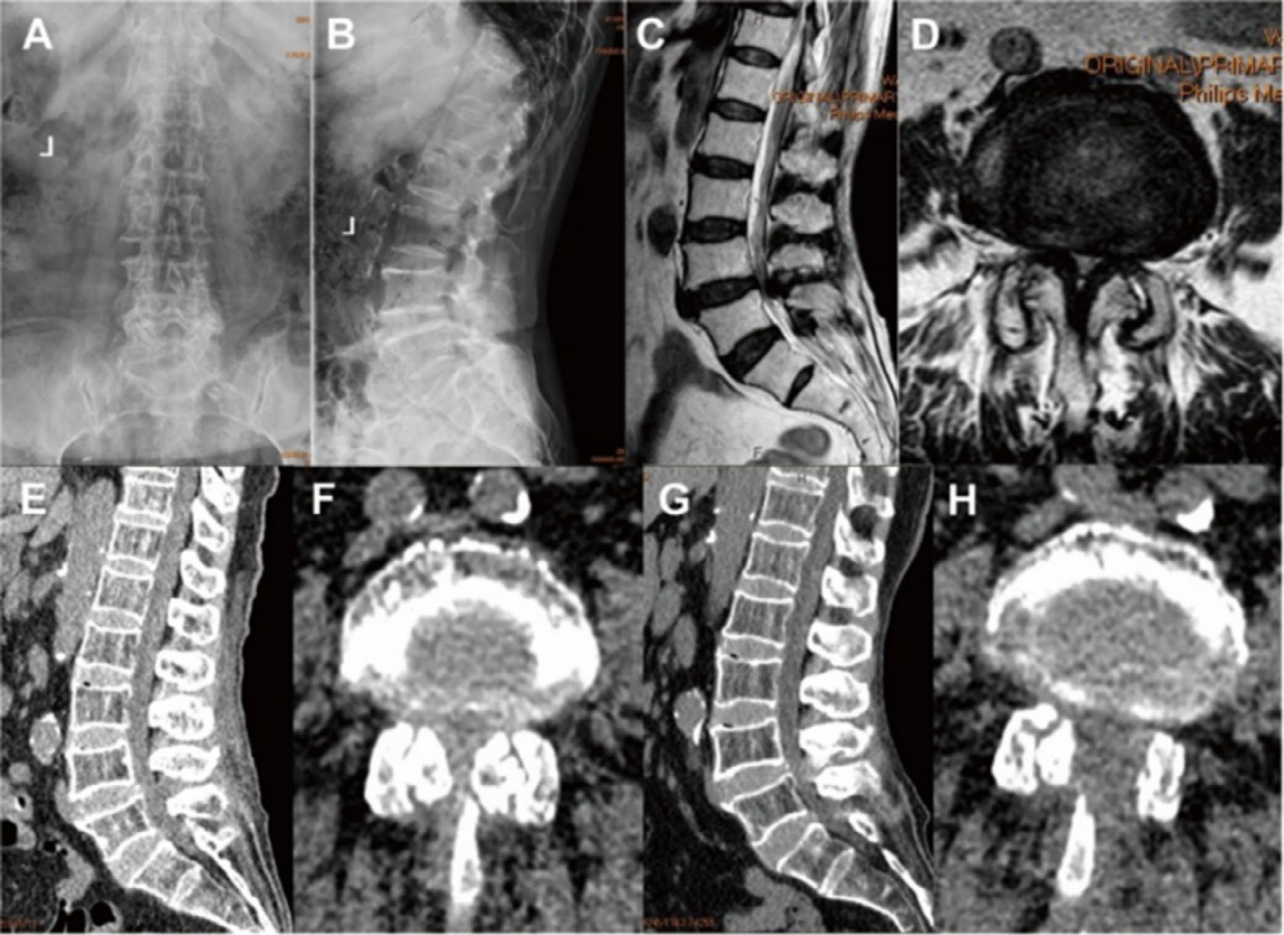
Radiological images of UBE technique under LA. (A, B) Preoperative AP and lateral view X rays showed obvious degeneration at the L4/5 level. (C–F) Preoperative MRI and CT scans showed stenosis at the L4/5 level. (G–H) Postoperative CT scans showed sufficient decompression after UBE decompression. AP, anterior–posterior; CT, computed tomography; LA, local anesthesia; MRI, magnetic resonance image; UBE, unilateral biportal endoscopy.

All the detailed steps were illustrated in Figures 1 and 2.

6 *Drainage placement and incision closure*.

### 2.3 Demographic Data Collection, Functional and Radiological Assessment

Demographic data including sex, age, medical comorbidities, American Society of Anesthesiologists (ASA) classification, surgical level, operation time, estimated blood loss, duration of postoperative hospitalization, and complications were systematically collected. Visual analog scale (VAS) scores for leg pain were evaluated preoperatively, immediately postoperatively, at the 3‐month follow‐up, and at the final follow‐up. The oswestry disability index (ODI) was assessed preoperatively, at the 3‐month follow‐up, and at the final follow‐up. The modified MacNab criteria were recorded at the final follow‐up. Anterior–posterior and lateral view X‐rays, computed tomography (CT) scans, and magnetic resonance images (MRI) of the lumbar spine were obtained preoperatively. Postoperative CT scans of the lumbar spine were reviewed after removal of the drainage tube.

### 2.4 Statistical Analysis

The Shapiro–Wilk test was used to evaluate the distribution of the collected data. Categorical variables were grouped and presented as numerical values, whereas normally distributed continuous variables were presented as *mean ± standard deviation*. Data comparisons between the preoperative, postoperative, and follow‐up time points were conducted using paired sample *t*‐tests. Statistical significance was defined as *p* < 0.05. All data analyses were performed using the SPSS v25.0 software (IBM Corp., Armonk, NY, USA).

## 3 Results

### 3.1 Demographic Data

Eighteen patients with a mean age of 77.1 ± 5.0 years were enrolled in this study, comprising 5 males and 13 females. Fifteen patients (83.3%) had medical comorbidities including hypertension, diabetes mellitus, coronary heart disease, and a history of cerebral infarction. Based on the ASA classification for anesthesia assessment, 14 patients (77.8%) were classified as Grade III, and four patients (22.2%) were classified as Grade II. Among the 18 surgical levels, 1 (5.6%) was at L3–4, 15 (83.3%) at L4–5, and 2 (11.1%) at L5–S1. Sixteen patients (88.9%) underwent unilateral decompression due to radiating leg pain on one side, two patients (11.1%) underwent bilateral decompression due to neurogenic claudication, and one patient (5.6%) underwent decompression combined with discectomy due to significant disc herniation. The mean duration of surgery, estimated blood loss, and postoperative hospitalization were 68.6 ± 22.0 min, 32.7 ± 10.7 mL, and 2.3 ± 0.5 days, respectively. The mean follow‐up period was 14.8 ± 7.9 months. Detailed data are listed in Table 1.

**TABLE 1:** Patients' information of UBE operation under LA.

No.	Gender	Age (years)	Diagnosis	Comorbidity	ASA	Operative level	Operation	Operative duration (min)	EBL (ml)	LOS (d)
1	F	74	LSS	None	III	L5/S1	Uni. decompression (R)	60	30	2
2	M	75	LSS	CHD	III	L4/5	Uni. decompression (L)	60	40	2
3	F	78	LSS	HTN, DM	III	L4/5	Bil. decompression	75	30	3
4	F	82	LSS	HTN	II	L4/5	Bil. decompression	60	20	2
5	F	71	LSS	HTN, DM, CRF	III	L5/S1	Uni. decompression (L)	60	60	2
6	F	77	LSS	HTN, CRF	III	L4/5	Uni. decompression (R) + discectomy	50	40	2
7	F	77	LSS	CHD, DM	III	L3/4	Uni. decompression (L)	60	30	3
8	M	80	LSS	HTN, CHD	III	L4/5	Uni. decompression (R)	85	30	3
9	F	76	LSS	HTN, CHD, CI	III	L4/5	Uni. decompression (R)	120	40	2
10	F	82	LSS	HTN, DM	II	L4/5	Uni. decompression (L)	90	50	2
11	F	74	LSS	DM, HF	III	L4/5	Uni. decompression (L)	70	30	2
12	F	78	LSS	None	II	L4/5	Uni. decompression (R)	60	40	2
13	M	75	LSS	CHD	III	L4/5	Uni. decompression (R)	120	20	3
14	F	76	LSS	HTN, CHD	III	L4/5	Uni. decompression (L)	45	20	3
15	M	65	LSS	HTN, CI, AVB	III	L4/5	Uni. decompression (R)	60	20	2
16	F	77	LSS	CHD, DM	III	L4/5	Uni. decompression (L)	50	30	3
17	M	89	LSS	HTN, DM, CI	III	L4/5	Uni. decompression (R)	50	30	2
18	F	82	LSS	None	II	L4/5	Uni. decompression (L)	60	30	2

Abbreviations: ASA, American Society of Anesthesiologists; AVB, atrioventricular block; Bil, bilateral; CHD, coronary heart disease; CI, cerebral infarction; CRF, chronic renal failure; DM, diabetes mellitus; EBL, estimated blood loss; HF, heart failure; HTN, hypertension; LA, local anesthesia; LOS, length of postoperative stay; LSS, lumbar spinal stenosis; UBE, unilateral biportal endoscopy; Uni, unilateral.

### 3.2 Clinical Outcomes of UBE Surgery Under LA

The mean VAS score for leg pain improved significantly immediately postoperatively (0.9 ± 0.6 vs. 5.8 ± 1.0, *p* < 0.001), at 3 months postoperatively (0.7 ± 0.6 vs. 5.8 ± 1.0, *p* < 0.001), and at the last follow‐up (0.5 ± 0.7 vs. 5.8 ± 1.0, *p* < 0.001), compared to the preoperative values. There were no significant differences in the VAS scores for leg pain between the immediate postoperative period, the 3‐month follow‐up, and the last follow‐up. The mean ODI score at 3 months postoperatively (21.1 ± 9.5 vs. 46.8 ± 11.3, *p* < 0.001) and at the last follow‐up (16.2 ± 7.2 vs. 46.8 ± 11.3, *p* < 0.001) showed clinically significant improvements compared to the preoperative ODI score. Moreover, the ODI score at the last follow‐up was significantly better than at the 3‐month follow‐up (16.2 ± 7.2 vs. 21.1 ± 9.5, *p* = 0.003). At the last follow‐up, based on the modified Macnab criteria, the following outcomes were observed: excellent in 13 patients (72.2%), good in one (5.6%), fair in two (11.1%), and poor in one (5.6%), yielding an excellent‐good rate of 77.8%. Three complications were observed during the follow‐up: one patient developed transient lower limb paralysis due to local anesthetic entering the subarachnoid space, which resolved spontaneously 6 h after surgery; the second patient's surgery was aborted due to acute onset of atrial fibrillation during the operation, and the third patient required reoperation for recurrent disc herniation 2 months after the primary UBE procedure. No cases of dural tear, neurological injury, or intraspinal hematoma were observed in any patient.

### 3.3 Patient Self‐Reported Pain During UBE Surgery Under LA

**FIGURE 3:**
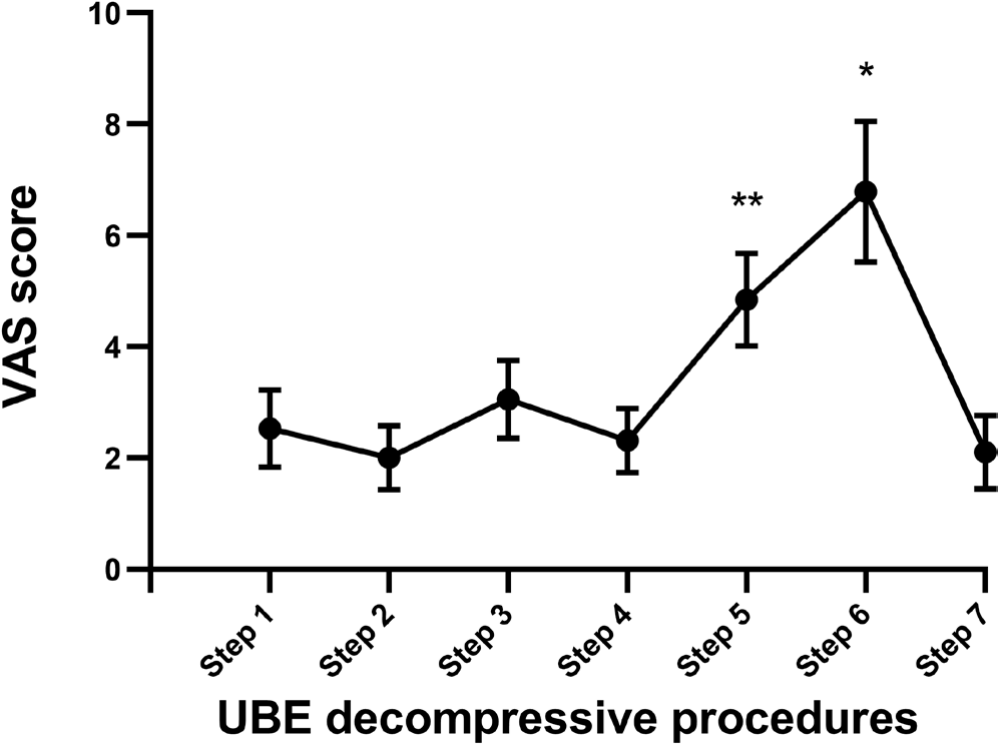
Patient self‐reported pain during UBE surgery under LA. The highest VAS score of intraoperative pain was at Step 6, followed by Step 5. LA, local anesthesia; UBE, unilateral biportal endoscopy; VAS, visual analog scale.

Intraoperative pain at the surgical site was assessed using VAS scores at each point of the seven‐step decompressive strategy during UBE surgery under LA. Among all seven steps, the highest VAS score for intraoperative pain was reported during the removal of the medial edge of the superior articular process and the lateral insertion point of the ligamentum flavum from *SafetyNet* (Step 6, mean 6.8 ± 1.2), followed by the removal of the distal insertion point of the ligamentum flavum from *SafetyNet* (Step 5, mean 4.8 ± 0.8). The self‐reported pain for Step 6 and 5 was significantly more severe than that of the other decompressive procedures (*p* < 0.001). However, none of the 18 patients voluntarily requested surgery termination because of unbearable intraoperative pain. The related data were illustrated in Figure 3.

## 4 Discussion

To our knowledge, this is one of the few studies that have investigated the feasibility and efficacy of UBE decompression for LSS under LA. Based on this study, satisfactory functional outcomes can be achieved by employing the *SafetyNet*‐guided decompression strategy, of which the excellent‐good rate is 77.8% based on the modified Macnab criteria. The *SafetyNet*‐guided decompression strategy employed in this study enables rapid and safe decompression of the spinal canal with good intraoperative pain tolerance.

### 4.1 Anesthesia Concerns for LSS Decompression Surgery Under GA or SA

GA is usually needed in most minimally invasive procedures for lumbar degenerative conditions, indicating the high risks associated with GA and the uncertainty of conducting the surgery, which was significantly influenced by perioperative evaluation by anesthesiologists [16, 25]. For elderly patients, minimally invasive spine surgery under GA may cause unignorable risk. One retrospective study analyzed the major and minor complications of minimally invasive surgery in 21 patients over 80 years of age, and the results showed that a complication rate of 66.7% and a mortality rate of 9.5% occurred within 30 days of surgery [26]. There have been applications of SA in minimally invasive spinal endoscopic surgeries [20, 21]. One meta‐analysis compared the complications associated with awake SA and GA in spinal surgery. Based on the data of 7820 patients from 38 studies, the overall complication, postoperative nausea/vomiting, and urinary retention rates were significantly higher in patients under GA than in those under SA [27]. However, for water‐mediated spinal endoscopic techniques such as UBE, SA still carries potential risks. Saline used for irrigation during the surgical procedure may enter the subarachnoid space through the SA puncture site, leading to increased intracranial pressure, subsequent seizures, and even death [22]. According to previous literature, a retrospective age‐matched case–control study conducted by Tae Hoon Kang et al. [28] compared three groups of patients (20 patients per group) who underwent UBE lumbar decompression under different anesthetic modalities: GA, SA, and erector spinae plane block (ESPB). In the ESPB group, all procedures were successfully completed without the need to convert to alternative anesthetic techniques and without any anesthesia‐related complications. Furthermore, the mean total length of hospital stay in the ESPB group was shorter compared to both the GA and SA groups. Notably, no cases of postoperative nausea and vomiting were observed in the ESPB group, even in the absence of prophylactic antiemetic treatment. In a retrospective study conducted by Tong Wu et al. [20], 31 consecutive patients underwent awake UBE decompression for degenerative lumbar spinal stenosis. Among them, 26 patients (83.9%) rated their intraoperative experience as satisfactory, categorized as either excellent or good, while five patients (16.1%) rated it as fair. At the final follow‐up, 28 patients (90.3%) reported the surgical outcome as good or excellent, and three patients (9.7%) rated it as fair. No serious complications or adverse reactions were observed throughout the study. Therefore, for UBE‐like water‐mediated spinal endoscopic surgery, total LA may theoretically have the advantage of reducing anesthesia‐related risks compared to SA.

### 4.2 Potential Advantages of SafetyNet‐Guided UBE Decompression Under LA

Given that uniportal endoscopy under LA is already widely practiced for treating LSS [23, 24], we attempted UBE decompression surgery under LA for LSS patients who were deemed by anesthesiologists to be at high risk for GA and achieved satisfactory clinical outcomes, as described above. We propose that UBE surgery under LA offers several technical advantages over GA. First, LA can significantly reduce the risks associated with GA, enhance surgical safety, lower the physiological burden on elderly patients, and prevent postoperative discomfort such as nausea and vomiting caused by GA, leading to faster recovery and shorter hospital stay. Additionally, during UBE surgery under LA, patients remain awake and can communicate with the surgeons, allowing them to receive real‐time feedback on changes in the patients' sensations and movements. This helps avoid potential procedures that might damage neural structures, further increasing the safety and effectiveness of the operation.

Based on this study, we believe that UBE decompression under LA is suitable for patients with single‐level LSS who primarily present with unilateral radiating leg pain. The *SafetyNet*‐guided decompression strategy employed in this study enables rapid and safe decompression of the spinal canal with good intraoperative pain tolerance, as confirmed by the patients' self‐reported pain assessments during the UBE procedure, as mentioned above. *SafetyNet* is an anatomical landmark located at the lower edge of the inferior articular process, at the base of the superior articular process. The fat pad surrounding the lower edge of the inferior articular process served as a marker for this region. Starting from *SafetyNet*, access to the spinal canal can be achieved by following the superior facet joint, the lower edge of the cephalic lamina, or the upper edge of the caudal lamina. The clinical significance of *SafetyNet* lies in the fact that its base is a solid cartilaginous surface of the superior articular process, which helps avoid damage to neural structures during spinal canal decompression. As demonstrated in this study, no dural tears or neurological injuries were observed in our case series, highlighting the potential anatomical value of *SafetyNet* in UBE decompression surgery for LSS.

In two specific cases (11.1%), bilateral decompressive procedures were performed. These patients presented with symmetrical bilateral neurological symptoms and severe spinal stenosis confirmed by MRI. Intraoperatively, following unilateral decompression, the patients—who were awake—continued to report contralateral symptoms. Consequently, decompression was extended to the contralateral side until symptom resolution was achieved. As the procedure was performed under local anesthesia with the patient fully conscious, intraoperative feedback could be obtained in real‐time to assess the adequacy of symptom relief. This allowed us to tailor the extent of decompression based on the patient's immediate responses. Consequently, during the postoperative follow‐up period, no patients required revision surgery due to insufficient decompression.

### 4.3 Considerations for UBE Procedure Under LA

However, unlike the traditional UBE procedure under GA, some issues with UBE under LA must be mentioned based on our initial experience. First, whether the patient could tolerate the prone position was a crucial factor for successful implementation of UBE under LA. Therefore, prior to surgery, we dedicate substantial time to thoroughly communicate procedural details with patients and conduct a 30‐min prone positioning training session in the ward, during which vital signs are closely monitored. Only patients who demonstrate adequate understanding and successfully complete this preoperative training are selected to undergo UBE surgery under local anesthesia. Additionally, the cases included in this study were conducted after one surgeon had proficiently mastered UBE decompression surgery, minimizing the duration of the patient's prone position to enhance their tolerance to the surgery. Second, radiofrequency coagulation cannot be used for hemostasis, especially in cases of epidural bleeding. This is because radiofrequency coagulation for hemostasis near the nerve roots may cause severe radiating pain in patients, potentially affecting their tolerance for UBE under LA and compromising the overall safety of the operation. Therefore, the surgical field may not be as clear as that under GA, which could affect decompression. We believe that using the attachment point of the ligamentum flavum as a marker for adequate bony decompression and achieving on‐bloc resection of the ligamentum flavum can prevent incomplete decompression effectively. In this study, no case required reoperation due to inadequate initial decompression. Third, resecting the medial edge of the superior articular process and exposing the lateral attachment point of the ligamentum flavum are key aspects of spinal canal decompression and major pain stage during UBE under LA, especially for patients with severe stenosis. Conventional procedures often use a Kerrison rongeur to resect the medial edge of the superior articular process from the inside out, compressing the nerve root and exacerbating obvious intraoperative pain. In contrast, using an ultrasonic bone scalpel allowed for resection of the superior articular process from the outside, avoiding further compression of the nerve root, and significantly reducing intraoperative pain. Moreover, studies have found that the use of an ultrasonic bone scalpel during surgery helps reduce intraoperative bleeding [29, 30].

Based on the results of our analysis, the highest pain scores during UBE under local anesthesia were observed at Step 5 and Step 6 of the procedure. In response to these findings, and following confirmation through this study, we implemented a clinical modification strategy in collaboration with the anesthetic monitoring team. Specifically, we administer an ultra‐short‐acting intravenous analgesic, remifentanil, immediately after the completion of Step 4 to mitigate the anticipated increase in pain. This adjustment represents a clinically applicable strategy directly guided by the outcomes of our study.

### 4.4 Limitations

However, this study has several notable limitations. First, the retrospective nature of this study, combined with its relatively small sample size, limits the generalizability of its findings and restricts the ability to perform more rigorous statistical analyses. Although the initial results are promising, a larger sample size and prospective data collection would better validate these findings and allow broader application. Second, only two patients underwent bilateral decompression, limiting insights into the feasibility and outcomes of LA‐based UBE in cases with more extensive stenosis. This limits the applicability of UBE under LA to only selected patient subsets and does not fully address the broader population of patients with LSS who may require bilateral decompression. Third, the absence of a general anesthesia (GA) control group within the same patient population limits the ability to contextualize and directly compare the efficacy and safety outcomes of the procedure. Finally, the study's follow‐up period of approximately 14.8 months provides limited information on the long‐term durability of the outcomes achieved through UBE under LA. Longer follow‐up periods are necessary to understand whether these initial improvements in pain and functionality are sustained and to assess potential delayed complications.

## 5 Conclusions

UBE decompression under LA is feasible in elderly patients with LSS and significant medical comorbidities. Satisfactory clinical outcomes can be achieved by employing the *SafetyNet*‐guided decompression strategy during UBE surgery under LA, making it a potential alternative treatment, especially for patients with LSS who are intolerant to GA.

## Author Contributions

**Haining Tan:** methodology, investigation, writing – original draft. **Yuquan Liu:** methodology, investigation, writing – original draft. **Guangpeng Li:** methodology, investigation, writing – original draft. **Lingjia Yu:** formal analysis, data curation. **Haibo Sun:** formal analysis, data curation. **Bin Zhu:** formal analysis, validation. **Qi Fei:** visualization, validation. **Yong Yang:** conceptualization, methodology, writing – review and editing. **Yuan‐Shun Lo:** visualization, validation. **Xiang Li:** conceptualization, methodology, writing – review and editing, supervision, project administration, resources.

## Disclosure

The authors have nothing to report.

## Ethics Statement

The study was conducted in accordance with the Declaration of Helsinki and approved by the Medical Ethics Committee of Beijing Friendship Hospital (BFHHZS20250021).

## Consent

Written informed consent to participate in the study was obtained from each participant before the study started.

## Conflicts of Interest

The authors declare no conflicts of interest.
